# Role of Women's Empowerment in Improving the Nutritional Status of Children Under Five Years of Age: An Insight From the National Family Health Survey-5

**DOI:** 10.7759/cureus.59410

**Published:** 2024-04-30

**Authors:** Gaurav Singh, Anupriya Jha

**Affiliations:** 1 Department of Community & Family Medicine, All India Institute of Medical Sciences, Raipur, Raipur, IND

**Keywords:** under-five children, maternal autonomy, undernutrition, empowerment, underweight, wasting, stunting, malnutrition, women’s empowerment

## Abstract

Background

Childhood malnutrition remains a global concern, especially in low- and middle-income countries, and is known to create an intergenerational cycle of illness and poverty. Women's empowerment has gained global recognition as a potential catalyst for improving child nutrition. The objective of this research was to investigate the association between women's empowerment factors and the nutritional status of children under five years of age.

Methods

The study used data from the National Family Health Survey-5, conducted in India between 2019 and 2021 by the International Institute of Population Sciences, Mumbai. A weighted sample of 29,491 mother-child pairs was analyzed. The odds ratio for women's empowerment and sociodemographic factors associated with the nutritional status of children under five years of age was calculated using Pearson’s chi-square test and multiple logistic regression.

Results

The study found that the sex of the child (OR = 1.066, 95% CI: 1.017 to 1.117; p-value: 0.008), birth order (OR = 0.824, 95% CI: 0.780 to 0.871; p-value < 0.001), education of the mother (OR = 1.356, 95% CI: 1.255 to 1.464; p-value < 0.001), education of the father (OR = 1.227, 95% CI: 1.140 to 1.320; p-value < 0.001), having a bank or savings account that she uses (OR = 1.151, 95% CI: 1.084 to 1.221; p-value < 0.001), having a mobile phone that she uses (OR = 1.184, 95% CI: 1.125 to 1.246; p-value < 0.001), and wealth index (OR = 1.597, 95% CI: 1.514 to 1.684; p-value < 0.001) were significant predictors of undernutrition in children under the age of five (U5).

Conclusion

Women's empowerment factors play a significant role in improving childhood nutrition. In the study, male sex, higher birth order, lower parental education, poor wealth index, maternal lack of access to a bank or a savings account, and lack of mobile phone ownership were found to be significant predictors of undernutrition in children U5. The findings underscore the importance of family planning, financial inclusion, access to mobile phones, and higher education for women as important factors that can help improve the nutritional status of children under five years of age. Policymakers can utilize these insights to make targeted strategies for women's empowerment, thus improving the nutritional status of children. However, being a secondary data analysis, our research is constrained by the variables available in the dataset. Further research is required to better comprehend the complex relationship between women empowerment and child nutrition.

## Introduction

Undernutrition is a prevalent problem that impacts approximately one-third of children worldwide and is a leading cause of illness and death in countries with low and middle incomes [1]. Undernutrition is defined as an insufficient intake of energy and nutrients, which is unable to meet an individual's requirements necessary for maintaining good health. The three most common anthropometric measures used to classify undernutrition are weight-for-age, height-for-age, and weight-for-height. We classify low weight-for-age, low height-for-age, and low weight-for-height as underweight, stunted, and wasted, respectively [2]. The World Health Organization (WHO) reported that wasting affected approximately 45 million children under the age of five (U5) in 2020, while stunting affected 149 million children worldwide. Undernutrition was found to be a factor in about 45% of deaths among children U5 [3,4]. Inadequate maternal and child care is a primary factor contributing to malnutrition in children. The influence of women's empowerment on child development and growth is widely recognized to be substantial [5-8].

The 2020-2030 Nutrition Strategy of the United Nations International Children's Emergency Fund (UNICEF), as outlined in their Framework on Maternal and Child Nutrition, highlights the connection between women's empowerment and the outcomes of children [3,8]. A woman who is empowered has the necessary skills and knowledge to make decisions within her household. This includes making informed choices regarding the best health practices and seeking appropriate medical and mental health care when needed [1,9]. Taking an active role in healthcare decisions empowers women to access appropriate care for their children and make use of prenatal and postnatal services. Moreover, being involved in family planning can result in enhanced nutritional outcomes for children by assisting mothers in controlling the size of their family and the timing of births [6]. The Sustainable Development Goals (SDGs) have established two aims: First, to guarantee that by 2030, all deaths of newborns and children U5 are preventable; second, to ensure that women have equitable access to leadership positions in public, political, and economic domains at all levels of decision-making in all countries [4,10]. Given this context, the research aimed to investigate the association of different factors related to women's empowerment and sociodemographic factors with the occurrence of undernutrition in children U5.

## Materials and methods

Source of data

The International Institute for Population Sciences, Mumbai, conducted the National Family Health Survey-5 (NFHS-5) during the years 2019-2021. NFHS-5 is a nationally representative cross-sectional survey. The survey employed a sampling technique known as two-stage stratified cluster sampling. The survey gathers data on diverse aspects of health, encompassing child nutrition and women's empowerment. The NFHS provides superior-quality, reliable health data. The NFHS-5 survey gathered data from a total of 636,699 households, which included 724,115 females and 101,839 males. This study utilized the birth recode (BR) dataset, which contains information on various parameters of women's health as well as the health status of children U5. The analysis included a weighted sample of 29,491 pairs of mothers and children after eliminating any missing data on the included study's variables. Further information on the NFHS-5 survey methodology can be found in another source [11]. The authors obtained authorization to utilize the data from the ICF-DHS program.

Dependent variable

Researchers categorized the dependent variable as the presence or absence of undernutrition in children U5. The assessment of undernutrition in children was based on the presence of any of the following conditions: wasting, stunting, or being underweight. In NFHS-5, health workers measured the height of children aged 24-59 months using the Seca 213 stadiometer and an infantometer for those under two years old. The assessment of underweight, wasting, and stunting involved comparing weight-for-age, weight-for-height, and height-for-age with standardized Z-scores. A Z-score below the -2 standard deviation indicated the presence of underweight, wasting, and stunting, respectively.

Independent variable

We have classified the independent variables into two categories: sociodemographic factors and factors associated with women's empowerment. Sociodemographic factors encompass various aspects such as the mother's age, birth order of the child, religion, place of residence, maternal education, paternal education, family's wealth index, child's gender, and child's age. We have conducted an assessment of women's empowerment based on eight distinct variables. These variables encompass women's involvement in household decision-making, ownership of a house or land either individually or jointly, possession of a personal bank or savings account, ownership and use of a personal mobile phone, freedom to independently visit the market, freedom to independently visit the health facility, and freedom to independently visit outside the village. These factors that contribute to women's empowerment can be grouped into decision-making authority within the family, financial autonomy, and freedom of mobility.

Statistical analysis

We conducted a bivariate analysis using the Chi-square test to determine the association between undernutrition (low weight-for-age, height-for-age, weight-for-height, respectively) occurrence in children U5, sociodemographic factors, and women's empowerment factors. The factors that were found to be statistically significant on bivariate analysis (p-value ≤ 0.05) were included in the multiple logistic regression analysis to identify the factors predicting undernutrition in children U5. We conducted the statistical analyses using SPSS, version 20 (IBM Corp., Armonk, NY).

## Results

The study included a weighted sample of 29,491 mother-child pairs. The average age of the mothers was 26.95 ± 4.841 years. The majority of women (n = 21885, 74.2%) resided in rural areas, identified as Hindu (n = 23300, 79.0%), and either had no formal education (n = 6073, 20.6%) or had completed education up to the secondary level (n = 18791, 63.7%). Approximately 50% (n = 13633, 46.3%) of the women were members of households classified as poor or poorest. The average age of the children was 29.95 ± 17.14 months. The distribution of male (n = 15279, 51.8%) and female (n = 14212, 48.2%) children was nearly equal.

Association between the occurrence of undernutrition in children aged five and below and different sociodemographic factors

Table 1 displays the association between undernutrition in children U5 and different sociodemographic factors. The sociodemographic characteristics that were found to have a statistically significant association with undernutrition in children U5, based on bivariate analysis, are as follows: age group of the mother (p-value = 0.015), place of residence (p-value < 0.001), religion (p-value < 0.001), wealth index of the family (p-value < 0.001), age group of the child (p-value = 0.004), birth order of the child (p-value < 0.001), mother's education (p-value < 0.001), father's education (p-value < 0.001), and sex of the child (p-value = 0.006).

**Table 1:** Association between undernutrition in children under five years of age and various sociodemographic factors

Sociodemographic variables		Total number (n = 29491)	Number of children with undernutrition (%)	p-value (Chi-square)
Age group (mother)	15-19 years	729	414 (56.8)	0.015
	20-34 years	26389	13557 (51.4)	
	35-49 years	2373	1214 (51.2)	
Place of residence	Rural	21885	11820 (54.0)	<0.001
	Urban	7606	3365 (44.2)	
Religion	Hindu	23299	11998 (51.5)	<0.001
	Muslim	4882	2636(54.0)	
	Others	1310	551 (42.1)	
Age group of children	0-6 months	3149	1540 (48.9)	0.004
	7-24 months	8736	4573 (52.3)	
	25-60 months	17607	9073 (51.5)	
Birth order	≤2	21584	10548 (48.9)	<0.001
	>2	7908	4637 (58.6)	
Wealth index	Poor	13633	8252 (60.5)	<0.001
	Middle	5871	2934 (50.0)	
	Rich	9988	4000 (40.0)	
Father education	Till secondary education	24566	13273 (54.0)	<0.001
	Higher than secondary education	4926	1913 (38.8)	
Mother education	Till secondary education	24864	13488 (54.2)	<0.001
	Higher than secondary education	4627	1697 (36.7)	
Sex of the child	Male	15279	7985 (52.3)	0.006
	Female	14212	7200 (50.7)	

Table 2 displays the association between undernutrition in children U5 and different factors related to women's empowerment. The statistically significant women empowerment factors associated with the presence of undernutrition in children U5 were having a bank account that she uses (p-value < 0.001), having a mobile phone that she uses (p-value < 0.001), owning a house alone or jointly (p-value < 0.001), and owning a land alone or jointly (p-value < 0.001).

**Table 2:** Association between women empowerment factors and undernutrition in children under five years of age

Women's empowerment factors		Total number (n = 29491)	Number of children with undernutrition (%)	p-value (Chi-square)
Usually allowed to go to the market	Not at all or with someone	15232	7906 (51.9)	0.142
	Can go alone	14259	7279 (51.0)	
Usually allowed to go to the health facility	Not at all or with someone	15983	8298 (51.9)	0.112
	Can go alone	13509	6888 (51.0)	
Usually allowed to go to places outside the village	Not at all or with someone	16632	8637 (51.9)	0.086
	Can go alone	12859	6548 (50.9)	
Do you have a bank account that you use?	Yes	23443	11772 (50.2)	<0.001
	No	6048	3413 (56.4)	
Do you have a mobile phone that you use?	Yes	17436	8243 (47.3)	<0.001
	No	12055	6942 (57.6)	
Are you involved in three major decisions of the household?	Yes	20005	10249 (51.2)	0.198
	No	9486	4936 (52.0)	
Do you own a house alone or jointly?	Yes	12491	6892 (53.3)	<0.001
	No	16551	8294 (50.1)	
Do you own land alone or jointly?	Yes	10156	5480 (54.0)	<0.001
	No	19335	9705 (50.2)	

Logistic regression was performed, incorporating significant factors (p-value ≤ 0.05) from the bivariate analysis. We checked the assumption before applying logistic regression. The assumption of multicollinearity was met. The logistic regression analysis yielded a significant result (p-value < 0.001). The goodness-of-fit test using the Hosmer-Lemeshow test indicated a p-value of 0.146, suggesting no significant lack of fit. Additionally, we found the Nagelkerke R^2^, which measures the proportion of explained variance in the model, to be 0.055.

Table 3 presents the results of the multiple logistic regression analysis. Sex of the child (OR = 1.066, p-value = 0.008), birth order (OR = 0.824, p-value < 0.001), education of the mother (OR = 1.356, p-value < 0.001), education of the father (OR = 1.227, p-value < 0.001), having a bank or savings account that she uses (OR = 1.151, p-value < 0.001), having a mobile phone that she uses (OR = 1.184, p-value < 0.001), and wealth index (OR = 1.597, p-value < 0.001) were found to be significant predictors of undernutrition in children U5.

**Table 3:** Predictors of undernutrition under five years of age identified by multiple logistic regression β: Regression coefficient; SE: Standard error; CI: Confidence interval; p-value: Probability value.

Predictors		β	SE	Adjusted odds ratio (95% CI)	p-value
Sex of the child	Male	0.064	0.024	1.066 (1.017-1.117)	0.008
	Female	Reference			
Birth order	≤2	-0.193	0.028	0.824 (0.780-0.871)	<0.001
	>2	Reference			
Education of the mother	Till secondary education	0.304	0.039	1.356 (1.255-1.464)	<0.001
	Higher than secondary education	Reference			
Education of the father	Till secondary education	0.204	0.037	1.227 (1.140-1.320)	<0.001
	Higher than secondary education	Reference			
Do you have a mobile that you use?	No	0.169	0.026	1.184 (1.125-1.246)	<0.001
	Yes	Reference			
Do you have a bank/savings account that you use?	No	0.140	0.030	1.151 (1.084-1.221)	<0.001
	Yes	Reference			
Wealth index	Poorest/Poor	0.468	0.027	1.597 (1.514-1.685)	<0.001
	Middle/Rich/Richest	Reference			

## Discussion

Mother’s role in the nutrition status of children is accepted globally. This study can help in better comprehending the role of women’s empowerment in improving the nutritional status of children U5. The study relies on a nationally representative dataset, which enhances its external validity.

The findings of this research demonstrate a significant association between maternal empowerment and undernutrition status. Various studies conducted globally and within India [3,5,7,12] corroborate this assertion. Prior studies conducted in Bangladesh, Pakistan, and India have established a positive association between enhanced childhood nutrition and the degree of empowerment experienced by women [13-16].

Mothers' level of education significantly influences their empowerment. Our research suggests that a higher level of maternal education is associated with a lower risk of childhood undernutrition. Paternal education also exhibits a similar association. Education enables parents to acquire precise information regarding healthcare and to facilitate their own and their children's healthcare access, thereby leading to enhanced child nutrition. Additional research further corroborates this [3,16,17]. Studies conducted in India have yielded similar results [10,15,18,19].

The current study found that female children had comparatively lower odds of undernutrition than male children. Numerous studies have documented the existence of gender bias, wherein male children receive preferential treatment [20]. Consequently, numerous studies have reported higher odds of undernutrition among female children [21]. Our study findings contradict this. Various studies conducted in Africa and Asia have shown results similar to ours [17, 22]. Many such studies have proposed a range of possible reasons, spanning from biological to societal contexts. However, it is important to note that all of these reasons are speculative [23]. Additional investigation is necessary to comprehensively comprehend the intricate interplay between societal and biological contexts.

We found a significant association between a higher birth order and higher odds of undernutrition in children. Other studies [10,24-25] have obtained similar results. The possible reason for higher odds of undernutrition with a higher birth order could be due to reduced parental attention and care, leading to a decline in prenatal and postnatal care as well as child checkups. An alternative explanation could be that an increase in the number of births occurring within a household is associated with a decrease in the distribution of food and resources within the household. As a consequence, neonates of higher birth order may encounter malnourishment alongside various other health hazards.

The current study found higher odds of undernutrition in children U5 who belonged to families with a lower wealth index as compared to those children who belonged to families with a higher wealth index. Household wealth serves as an indicator of the economic status of a household. Enhanced prosperity reduces the probability of child undernutrition by allowing households to allocate funds for their children's improved nutritional requirements. Other studies [3,16,17,26] have reported similar results.

Our study revealed that indicators related to women's empowerment, such as personal mobile phone ownership and personal bank or savings account ownership, had a significant impact on reducing the odds of undernutrition compared to women without mobile phones and bank or savings accounts. These two factors contribute to the attainment of financial autonomy, thereby empowering women to make informed decisions regarding their access to medical and mental health services, as well as family planning. Other studies corroborate this finding, determining that financial inclusion mitigates child malnutrition by empowering disadvantaged and impoverished individuals to effectively address difficult circumstances like child malnutrition. Easy access to funds empowers individuals to provide necessary financial support for their children [26-28]. It also leads to enhanced decision-making capabilities, prompt implementation of measures for the well-being of children, acknowledgment of optimal health practices, and enhanced caregiving [1,5].

In our study's bivariate analysis, we discovered that children U5 whose mothers own a house, either alone or jointly, or own land alone or jointly, have higher odds of undernutrition compared to children whose mothers do not own these properties. However, further analysis using logistic regression revealed that the mother's ownership status of houses and land acted as confounders, and these factors did not significantly predict undernutrition in children U5.

Despite the above-mentioned findings, the logistic regression model (Nagelkerke R2 0.055) indicates that there are additional factors that could potentially influence children's nutritional status; however, because our research relies on secondary data analysis, we are unable to evaluate any additional factors such as access to healthcare services, family planning, personal childcare practices, mother-child dietary patterns, past disease exposures, and the use of maternity care services that could have an impact on our findings. The cross-sectional nature of the data prevents us from establishing a direct link between nutrition for children and mother empowerment. Data related to women's empowerment was self-reported in nature, which makes our findings vulnerable to social desirability bias.

## Conclusions

In India, undernutrition in children U5 is a major public health problem. Our study's findings shed light on the critical link between women's empowerment and undernutrition in children U5. We found that the significant predictors of undernutrition in children U5 include being a male child, having a higher birth order, having lower parental education, having a poor wealth index, the mother's lack of access to banking services, and the mother's lack of mobile phone ownership. Women, who are the primary caregivers in the family, are responsible for the child's care. Programs focusing on family planning, increased women's education, financial inclusion through bank account opening, and increased access to information and communication technology, such as mobile phones and digital literacy, can empower women to address nutritional challenges in children. Women's empowerment acts as an enabling factor for better child nutrition. Moving forward, further research is required to comprehensively comprehend the complex relationship between women's empowerment and the nutritional status of children.
